# Isolation and Characterization of *Komagataeibacter piraceti* sp. nov. and *Novacetimonas labruscae* sp. nov.: Two Novel Microaerobic Cellulose-Producing Acetic Acid Bacteria from Vinegars

**DOI:** 10.3390/microorganisms13020456

**Published:** 2025-02-19

**Authors:** Bernarda Karničnik, Tomaž Accetto, Lijana Fanedl, Igor Jugović, Janja Trček

**Affiliations:** 1 Department of Biology, Faculty of Natural Sciences and Mathematics, University of Maribor, 2000 Maribor, Slovenia; bernarda.karnicnik1@gmail.com (B.K.); igorjugovic444@gmail.com (I.J.); 2 Department of Microbiology, Biotechnical Faculty, University of Ljubljana, 1000 Ljubljana, Slovenia; tomaz.accetto@bf.uni-lj.si (T.A.); lijana.fanedl@bf.uni-lj.si (L.F.)

**Keywords:** Acetic acid bacteria, *Acetobacteraceae*, *Komagataeibacter*, *Novacetimonas*, *Komagataeibacter piraceti*, *Novacetimonas labruscae*, vinegar

## Abstract

The genera *Komagataeibacter* and *Novacetimonas* comprise industrially important species that produce various foods, nanocellulose, acetan-like polysaccharides, enantioselective sugars, and other valuable products. Here, we describe two novel strains, Hr1 and Jurk4, isolated from pear and apple-grape organic vinegars that showed very high (≥99.39%) 16S rRNA gene sequence identities to species of the *Komagataeibacter* and *Novacetimonas* genera, respectively. However, analysis of the 16S-23S rRNA gene internal transcribed spacer (ITS) sequences revealed only 92.6% sequence identity between the Hr1 strain and its closest relative, *Komagataeibacter sucrofermentans* LMG 18788^T^, and 93.8% sequence identity between the Jurk4 strain and its closest relative, *Novacetimonas cocois* JCM 31140^T^. Further whole-genome analysis showed for both strains an average nucleotide identity (ANI) below 94% and an in silico DNA–DNA hybridization (dDDH) value of less than 70% to their closest species, supporting their distinction as novel species. The strain Hr1 can be phenotypically differentiated from its closest *Komagataeibacter* species based on its ability to utilize (NH_4_)_2_SO_4_ as the sole nitrogen source in Asai medium with D-glucose and its inability to grow with 1-propanol as a sole carbon source. The strain Jurk4 can be differentiated from other *Novacetimonas* type strains based on its ability to produce 5-keto-D-gluconic acid, its growth in a medium with glycerol as the sole carbon source, and its inability to grow in an Asai medium with D-glucose. Both strains produce cellulose and possess clusters for acetane-like polysaccharide production, although of different types, which makes them industrially relevant. Based on these findings, we propose *Komagataeibacter piraceti* sp. nov. Hr1^T^ (=ZIM B1167^T^ = LMG 33628^T^) and *Novacetimonas labruscae* sp. nov. Jurk4^T^ (=ZIM B1166^T^ = LMG 33630^T^) as two novel members of the acetic acid bacteria group.

## 1. Introduction

The genera *Novacetimonas* and *Komagataeibacter* are taxonomically classified within the family *Acetobacteraceae* of the class α-*Proteobacteria* [1,2,3,4]. They represent 2 of the 25 genera that comprise the group known as acetic acid bacteria (AAB) [5,6,7,8,9,10]. The genus *Komagataeibacter* was first described in 2013 during the redefinition of the genus *Gluconacetobacter*, reclassifying the *Gluconacetobacter xylinus* clade into the newly established genus *Komagataeibacter* [3,11]. Species belonging to *Komagataeibacter* are characterized as non-motile, non-flagellated bacteria capable of producing acetic acid from ethanol and oxidizing acetate and lactate to water and carbon dioxide. They are unable to produce 2,5-diketo-D-gluconate from D-glucose [2]. This bacterial genus exhibits phenotypic diversity, including variations in carbon source utilization, bacterial nanocellulose production yields, strain stability, and pellicle structure [12]. In 2022, some species within the *Komagataeibacter* genus were reclassified into a novel genus, *Novacetimonas*, based on phylogenomic and comparative genomic analysis [1].

Members of the genera *Komagataeibacter* and *Novacetimonas* are particularly well known for their ability to synthesize cellulose, which is a highly valued material in industry due to its natural origin, biodegradability, stability, low toxicity, and distinctive viscoelastic properties [13,14,15,16]. Bacterial cellulose is a purer alternative to plant-based cellulose, as it is free of lignin, hemicellulose, and pectin [17,18]. This makes it a highly versatile material with significant potential. Its applications range from the food, packaging, and pharmaceutical industries to biomedical uses, including cosmetics, artificial skin for burns, etc. [19,20]. In addition to cellulose, many species from these genera can also produce water-soluble acetan-like polysaccharides that have potential applications in the food and pharmaceutical industries as natural additives [21,22]. Additionally, they exhibit antimutagenic and antioxidant properties [23]. Recently, sirtuin-like proteins have been identified in some AAB genomes [24]. These enzymes, known for their NAD^+^-dependent deacylation activity, play crucial roles across all domains of life [25]. Since sirtuins may participate in various metabolic pathways, a deeper understanding of their mechanisms could also lead to novel biotechnological applications in AAB [26,27].

Using a recently developed direct identification approach based on 16S-23S rRNA gene internal transcribed spacer (ITS) amplicon metagenomics for species identification of AAB [28], we identified several novel species of AAB in pear and apple-grape organic vinegars [29]. In this study, we describe the isolation and characterization of *Komagataeibacter* strain Hr1 from organic pear vinegar and *Novacetimonas* strain Jurk4 from organic apple-grape vinegar. Comprehensive phenotypic and genome analyses confirmed both strains as novel species. We propose the names *Komagataeibacter piraceti,* with Hr1^T^ as the type strain, and *Novacetimonas labruscae,* with Jurk4^T^ as the type strain.

## 2. Materials and Methods

### 2.1. Isolation of the Strains Hr1 and Jurk4

A self-sufficient farmer from the Pohorje region in Northeast Slovenia provided the vinegar samples. The samples were taken from the surface of the liquid in the barrels for vinegar production and transported to the laboratory in sterile flasks with three-hole-membrane (0.2 μm) screw caps (DWK Life Sciences, Mainz, Germany). They were diluted 10–1000-fold in 0.85% NaCl before being spread (0.1 mL) onto an RAE medium composed of glucose (40 g/L), peptone (10 g/L), yeast extract (10 g/L), citric acid (1.37 g/L), and Na_2_HPO_4_ × 2H_2_O (3.38 g/L), supplemented with 1% (*v*/*v*) ethanol and 1% (*v*/*v*) acetic acid. The plates were incubated for 3–7 days at 30 °C and high relative air humidity [30]. Morphologically different colonies were selected for strain isolation. The strain Hr1 was isolated from organic pear vinegar and Jurk4 was isolated from organic apple-grape vinegar. Isolates were stored in a liquid medium with 20% glycerol at −80 °C.

### 2.2. Analysis of Vinegar

The total acid content of both vinegars was determined through acid–base titration using 0.1 M NaOH and phenolphthalein as an indicator. The alcohol content was measured with an ebullioscope, which determines the boiling point of the sample and correlates it to alcohol concentration based on a reference scale. pH values were measured with a pH meter. Reducing sugars were analyzed using the 3,5-dinitrosalicylic acid (DNS) method, which involves the reaction of reducing sugars with a DNS reagent under heat, forming a colored complex measured spectrophotometrically [31]. A detailed analysis is provided in our previous paper [32].

### 2.3. DNA Sequencing

The isolates were inoculated from −80 °C on the RAE medium prepared as described above. A single colony was spread on RAE plates and incubated for three days to obtain enough biomass for DNA extraction. The primer pair SpaFw (5′-TGCGGCTGGATCACCTC-3′) and SpaRev (5′-GTGCCAAGGCATCCACC-3′) was used for the amplification of the 16S-23S rRNA gene ITS region. The PCR products were purified with a GenJet PCR Purification Kit (Thermo Scientific, Waltham, MA, USA) and sequenced by the Sanger method at Microsynth (Vienna, Austria). The Blast algorithm at NCBI was then used to analyze the sequences. 

For genome sequencing, biomass was sent to MicrobesNG (Birmingham, UK) for hybrid genome sequencing with Illumina and Nanopore technologies. For Illumina sequencing, genomic DNA libraries were prepared using the Nextera XT Library Prep Kit (Illumina, San Diego, CA, USA) following the manufacturer’s protocol. DNA quantification and library preparation were carried out on a Hamilton Microl STAR automated liquid handling system (Hamilton Bonaduz AG, Bonaduz, Switzerland). Libraries were sequenced on a lllumina NovaSeq 6000 (Illumina, San Diego, CA, USA) using a 250 bp paired-end protocol. Reads were adapter-trimmed using Trimmomatic version 0.30 [33] with a sliding window quality cutoff of Q15. For long-read sequencing, DNA libraries were prepared with Oxford Nanopore Technologies SQK-RBK114.96 kit (ONT, Oxford, UK) using 200–400 ng of high-molecular-weight DNA. Barcoded samples were pooled together into a single sequencing library and loaded into a FLO-MIN114 (R.10.4.1) flow cell in a GridION (ONT, UK). Hybrid assembly was performed using Unicycler version 0.4.0 [34], and contigs were annotated using Prokka version 1.11 [35]. The 16S rRNA gene sequences were extracted from the genomes.

16S-23S rDNA ITS amplicon metagenomics was carried out as described recently by Ribič and Trček [28].

### 2.4. Phenotypic Analysis

The phenotypic characteristics of the strains Hr1 and Jurk4 were compared with those of the closest type strains. The strain Hr1 was compared to the following strains from the genus *Komagataeibacter*: *Komagataeibacter melomenusus* AV436^T^, *Komagataeibacter nataicola* LMG 1536^T^, *Komagataeibacter kakiaceti* LMG 26206^T^, *Komagataeibacter sucrofermentans* LMG 18788^T^ and *Komagataeibacter xylinus* 1515^T^. The genus *Novacetimonas* currently comprises only five species, therefore the strain Jurk4 was compared with *Novacetimonas cocois* JCM 31140^T^, *Gluconacetobacter entanii* AV429, *Novacetimonas hansenii* LMG 1527^T^, *Novacetimonas pomaceti* T5K1^T^, and *Novacetimonas maltaceti* LMG 1529^T^. The type strain of the species *Gluconacetobacter entanii* is no longer cultivable. Therefore, the strain *Gluconacetobacter entanii* AV429, which has previously been proposed as a reference strain, was included in the analysis [36].

All tests were performed as previously described by Škraban et al. [37], except for gluconic acid identification, which was performed according to the modified method as described by Marič [38]. Growth in the presence of different concentrations of ethanol and acetic acid was tested in liquid RAE medium containing 1% ethanol and 2.5, 3, 4, 5, 6, and 7% of acetic acid or RAE medium containing 3% ethanol and 2.5, 3, 4, 5, 6, and 7% of acetic acid in test tubes. The cultures were incubated for seven days at 30 °C and 180 rpm. Growth in all tests conducted in a liquid medium was considered positive if the optical density at 600 nm was more than 0.2. An optical density between 0.07 and 0.2 indicated weak growth, while a value below 0.07 indicated no growth.

Additionally, the ability of Hr1 and Jurk4 to grow under microaerobic and anaerobic conditions was assessed. Both strains were cultivated on RAE medium supplemented with 1% ethanol and 1% acetic acid in microaerobic conditions (GENbox microaer, bioMérieux, Marcy l’Etoile, France) with an O_2_ concentration between 6.2 and 13.2% after 1 h of incubation and a CO_2_ concentration between 2.5 and 9.5% after 24 h of incubation. The anaerobic conditions were established with a commercial atmospheric generator bag (GENbox anaer, bioMérieux, Marcy l’Etoile, France), which supports an O_2_ concentration less than 0.1% after 2.5 h and a CO_2_ concentration higher than 15% after 24 h of incubation. The control group was cultivated in aerobic conditions. After incubation at 30 °C for five days, plates were checked for bacterial growth and subcultured altogether three times. Growth was considered present if the plate was covered with colonies too numerous to count and it was assessed as weak if colonies present on the medium were countable (<300).

The ability of the strains Hr1 and Jurk4 to form cellulose pellicles was tested in 50 mL RAE medium in 250 mL baffled Erlenmeyer flasks with three-hole-membrane (0.2 μm) screws (DWK Life Sciences). One medium-sized bacterial colony was inoculated on the growth medium, and cultures were incubated for 24 h at 180 rpm at 30 °C. The flasks were then incubated for an additional three days under static conditions at 30 °C. The cellulose pellicle was cooked in 0.5 M NaOH for 1 h at 80 °C. After processing, the membranes were neutralized in distilled water and air-dried.

### 2.5. Cellular Fatty Acid Analysis

The whole cellular fatty acids of the strains Hr1 and Jurk4 were determined from cultures grown on RAE medium supplemented with 1% ethanol and 1% acetic acid. Plates were incubated for three days at 30 °C under aerobic conditions. Inoculation, harvesting of the cells, and fatty acid methyl ester analysis were performed according to the recommendations of the commercial identification system MIDI (Sherlock Microbial Identification System, Inc., Newark, DE, USA). The fatty acid methyl esters were separated using gas chromatography (Agilent 6890, Santa Clara, CA, USA) and identified with the aerobe database RTSBA6 (Sherlock v, 6.1).

### 2.6. Antimicrobial Resistance

For the analysis of antimicrobial resistance, the disk diffusion method adapted from EUCAST guidelines was used. Briefly, strains were revitalized and precultured on RAE medium and incubated aerobically for three days at 30 °C and high relative air humidity. Afterward, the biomass was harvested and suspended in 0.85% NaCl, and the turbidity was adjusted to the value of the McFarland standard of 0.5. Using a sterile cotton swab, the bacterial suspension was then evenly applied over the surface of an RAE plate supplemented with 1% (*v*/*v*) ethanol and 1% (*v*/*v*) acetic acid. The antibiotic disks (BioRad, Hercules, CA, USA) were applied onto the media: 10 µg of gentamicin (GMN10), 10 µg of ampicillin (AMP10), 30 µg of chloramphenicol (CHL30), 5 µg of ciprofloxacin (CIP5), 15 µg of erythromycin (ERY15), and 5 µg of trimethoprim (TMP5). The diameter of the growth inhibition zone was measured after three days of incubation at 30 °C and at a high relative air humidity. The genomes of the strains Hr1 and Jurk4 were also analyzed for possible antibiotic resistance molecular determinants with an online Resistance Gene Identifier [39].

### 2.7. Bioinformatics

*Komagataeibacter* and *Novacetimonas* phylogenies were reconstructed using concatenated core genes using the maximum likelihood algorithm implemented in PhyML [40], with the GTR nucleotide substitution model and 1000 bootstrap replicates.

ANI values between publicly available genome sequences of the type strains and the strains Hr1 and Jurk4 were calculated using the software tool JSpecies [41]. Similarly, genome distances between publicly available genomes and the strains Hr1 and Jurk4 were calculated using the Genome-to-Genome Distance Calculator 3.0. This method reliably mimics conventional DNA–DNA hybridization [42].

The genomes of strains Hr1 and Jurk4 were analyzed using the Blastp algorithm to identify operons and gene clusters essential for the ecology of *Komagataeibacter* and *Novacetimonas*. The analysis focused on acetic acid resistance-conferring genes (*aarC*, *azr1*, *aatA*), alcohol and aldehyde dehydrogenase genes (*adhA*, *aldh*), and extracellular polysaccharide synthesis operons and gene clusters (*bcs*, *ace*). Prophage prediction was made with Cenote-Taker2 [43] and PHASTEST [44]. Additionally, sirtuin-like proteins were identified as described by Jugović and Trček [24] and were further analyzed using InterProScan [45].

## 3. Results and Discussion

### 3.1. Vinegar Characteristics and Isolation of Strains

**Figure 1:**
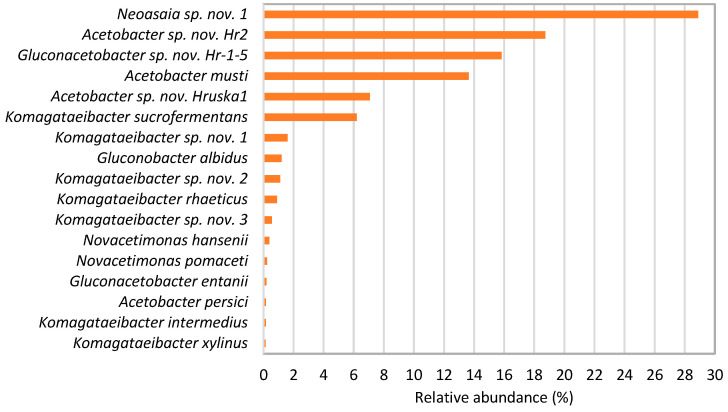
A relative abundance of AAB species in pear vinegar identified by comparing the 16S-23S rRNA gene ITS sequences to the reference strains deposited in the NCBI database. The sequences representing ≥ 0.1% of those obtained by ITS amplicon metagenomic analysis were considered. The species marked by sp. nov. showed very low ITS-sequence identity to the reference strains (≤95%).

**Figure 2:**
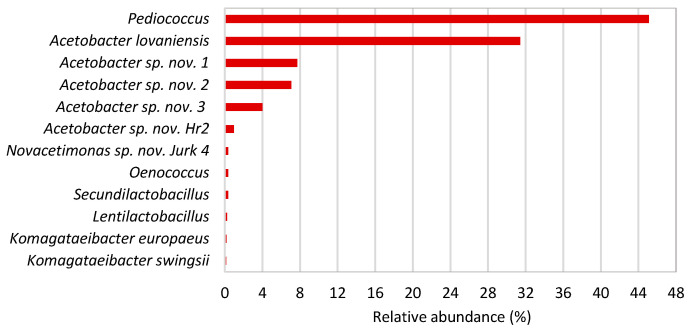
A relative abundance of AAB species in apple-grape vinegar identified by comparing the 16S-23S rRNA gene ITS sequences to the reference strains deposited in the NCBI database. The sequences representing ≥0.1% of those obtained by ITS amplicon metagenomic analysis were considered. The species marked by sp. nov. showed very low ITS-sequence identity to the reference strains (≤95%). *Novacetimonas* sp. nov. Jurk4 is also shown.

The basic chemical characteristics of both vinegars used as sources for isolating strains Jurk4 and Hr1 are presented in Table 1. Apple vinegar usually contains 3.9–9.0% acids and a maximum of 0.3% alcohol [46]. The analyzed apple-grape vinegar matches this description. In contrast, pear vinegar had a very low proportion of titratable acids, at only 0.54%. Moreover, it still contains 1.0% alcohol, indicating that the vinegar was in the formation process during sampling. This substrate creates a unique habitat for bacterial species and could support the presence of species not commonly found in vinegar. In addition to measuring the pH and determining the content of titratable acids and alcohol, we also checked for reducing sugars in both samples. While the apple-grape vinegar had no reducing sugars, the pear vinegar had a reducing sugar content of 3.8%. These reducing sugars may lead to a greater diversity and abundance of bacterial species in pear vinegar than in apple-grape vinegar. We confirmed that the examined pear vinegar exhibits greater species diversity and abundance than apple-grape vinegar (Figure 1 and Figure 2).

Nine acetic acid bacteria (AAB) species have been isolated from pear vinegar, mainly from the genus *Acetobacter*, and only three species from apple-grape vinegar (Table 2). Three strains from pear vinegar were identified as potential novel species from the genera *Komagataeibacter* (=*K. piraceti* Hr1), *Acetobacter*, and *Gluconacetobacter*, and one strain from apple-grape vinegar was identified as a potential novel *Novacetimonas* species (=*N. labruscae* Jurk4). Alongside obtaining a wide range of isolates from different species in pear vinegar, metagenomic analysis of 16S-23S rRNA gene ITS amplicons further confirmed its high diversity, with a total of 17 species identified (Figure 1). Interestingly, this analysis did not identify the presence of the strain Hr1. This is most likely due to its very low relative abundance, below 0.1%, which was set as the cut-off in bioinformatic analysis. Using the same amplicon metagenomics approach, eight species of AAB were identified in apple-grape vinegar, in addition to lactic acid bacteria, among which the genus *Pediococcus* was the predominant (Figure 2).

Apple-grape vinegar contained four additional potential novel species alongside the newly isolated *Novacetimonas labruscae* Jurk4 (Figure 2). All are from the genus *Acetobacter*, but isolation attempts were unsuccessful. One is *Acetobacter* sp. nov. Hr2, which we successfully isolated from pear vinegar. This species is present in both vinegars analyzed, which is reasonable since both were produced by the same farmer using the same bacterial starter culture. The predominant species in pear vinegar was from the genus *Neoasaia*, which we did not isolate. To date, this genus contains only a single species, *Neoasaia chiangmaiensis*, originally isolated from the flower of red ginger on a medium containing 0.3% acetic acid [47]. For the isolation of this genus, an RAE medium with a lower concentration of acetic acid than we used is likely necessary. Seven novel species were identified in pear vinegar, among which we successfully isolated three: *Acetobacter* sp. nov. Hr2, *Gluconacetobacter* sp. nov. Hr-1-5, and *Komagataeibacter* sp. nov. Hr1. The species *Acetobacter* sp. nov. Hruska1, indicated by the analysis of pear vinegar (Figure 1), was not isolated from the vinegar but was successfully isolated from the surface of the “vinska moštnica” pear variety in separate experiments. The presence of this species in pear vinegar is unsurprising, as the vinegar was produced from this pear variety. This finding also challenges the emerging assumption that all novel species found in this vinegar are primarily a result of speciation due to adaptation to specific conditions in vinegar, as some species are introduced to the vinegar from the fruits used for its production.

In addition, species were isolated from the vinegars that were not detected by the metagenomics of rDNA ITS amplicons, as only species present at more than 0.1% were included in the results. These species were *Komagataeibacter xylinus* and *Acetobacter aceti* from apple-grape vinegar and *Acetobacter aceti*, *Acetobacter ghanensis,* and *Acetobacter pasteurianus* from pear vinegar.

### 3.2. Morphological and Physiological Properties

Both the strains Hr1 and Jurk4 grew very well on RAE medium supplemented with 1% (*v*/*v*) ethanol and 1% (*v*/*v*) acetic acid, as well as on media without ethanol and acetic acid, such as glucose–yeast agar, mannitol agar, and RAE without acetic acid and ethanol. The strains failed to grow in a medium containing only 0.5% yeast extract (at pH 6.8), while both were capable of oxidizing ethanol to acetic acid and later to CO_2_ and H_2_O on Carr agar media, which is one of the typical characteristics of the genus *Komagataeibacter* [2]. The standard microbiological analysis showed that Hr1 and Jurk4 are Gram-negative, catalase-positive, and oxidase-negative.

Both strains showed moderate tolerance to acetic acid, with Hr1 growing strongly at a content of up to 3% acetic acid with 1% ethanol and 2.5% acetic acid with 3% ethanol. Jurk4 also grew well in a medium with up to 3% acetic acid with 1% ethanol and concentrations up to 3% acetic acid with 3% ethanol. The tolerance tests for different acetic acid concentrations would be expected to reveal greater differences between the two strains, as their substrates (the two vinegars) differ considerably in acidity and ethanol concentration. Thus, we anticipated that Hr1 would tolerate much lower concentrations of acetic acid (its natural habitat contains 1% (*v*/*v*) alcohol and 0.54% (*w*/*v*) titratable acids) compared to Jurk4 (whose habitat contains 0.3% (*v*/*v*) alcohol and as much as 3.84% (*w*/*v*) titratable acids). They may possess adaptable tolerance mechanisms; in addition, the controlled laboratory conditions do not perfectly replicate natural ones.

Besides growth under aerobic conditions, both Jurk4 and Hr1 exhibited the capability to tolerate microaerobic conditions, while none grew under anaerobic conditions. Some studies have already suggested that certain species within the AAB group can grow under lower oxygen concentrations—for instance, *Komagataeibacter xylinus*, *Gluconobacter* sp., and especially those AAB that have adapted to life in the gut of insects, where they are exposed to lower concentrations of O_2_ than those present in our atmosphere [48,49,50]. Due to their ability to grow even at low oxygen concentrations, they can cause spoilage. On the other hand, this trait could be helpful in industrial applications, where oxygen levels can fluctuate.

On RAE medium with 1% ethanol and 1% acetic acid, Hr1 formed round-shaped colonies around 0.5 mm in diameter, and Jurk4 formed irregularly shaped colonies around 0.5–1 mm in diameter after three days of incubation. The average length of the Hr1 cell was 2.35 µm, and the average width was 0.64 µm. Jurk4 cells had an average length of 3.02 µm and an average width of 0.89 µm.

### 3.3. Phylogenetic Analysis

It has previously been shown that sequencing 16S-23S rRNA gene ITS, which contains two highly conserved tRNA, is a reliable approach for identifying AAB species [51]. Comparative analysis of the 16S-23S rRNA gene ITS region revealed that *Novacetimonas cocois* JCM 31140^T^ is the nearest relative of Jurk4, with 93.84% identity, and *Komagataeibacter sucrofermentans* LMG 18788^T^ the nearest relative of Hr1, with 92.58% identity. Comparative analysis based on 16S rRNA gene sequences showed just a few nucleotide differences with their closest relatives. The strain Hr1 exhibited the highest 16S rRNA gene pairwise sequence identity to *Komagataeibacter xylinus* LMG 1515^T^ and *Komagataeibacter sucrofermentans* DSM 15973^T^, both at 99.44%. The closest strain to Jurk4 is *Novacetimonas hansenii* NCIB 8746^T^, with 99.39% sequence identity. Among the different species of AAB, 16S rRNA gene sequences can be very similar, which makes it impossible to classify the strains with certainty at the species level.

**Figure 3:**
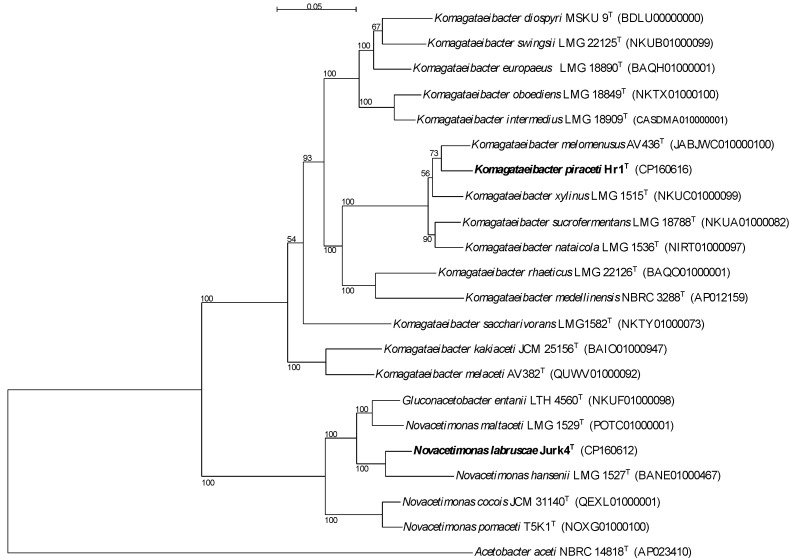
Phylogenetic reconstruction based on 46 identified core genes of the type strains of *Komagataeibacter* and *Novacetimonas* species. The tree was constructed using the maximum-likelihood method, with bootstrap values indicated at branching (1000 replicates). Genome accession numbers are presented in brackets. The scale bar represents 5% of estimated sequence differences.

After genome sequencing, a phylogenetic tree was constructed based on core genes (Figure 3). According to this phylogenetic tree, the closest type strains to Jurk4 and Hr1 were selected for the phenotypic characterization. The nearest relative of Hr1 is *Komagataeibacter melomenusus* AV436^T^, followed by *Komagataeibacter xylinus* LMG1515^T^, *Komagataeibacter sucrofermentans* LMG 18788^T^, and *Komagataeibacter nataicola* LMG1536^T^. In the genus *Novacetimonas*, there are only five species to date. Jurk4 is most closely related to *Novacetimonas hansenii* LMG1527^T^, followed by *Gluconacetobacter entanii* LTH4560^T^ and *Novacetimonas maltaceti* LMG1529^T^.

ANIb and ANIm analysis of the complete genomes of Hr1 and Jurk4 in comparison with the genomes of their nearest neighbors confirmed the results already given by the phylogenetic tree. Based on ANIb and ANIm (Table S1), Hr1 is most closely related to *Komagataeibacter melomenusus* AV436^T^, with an ANIb value of 92.62% and an ANIm value of 93.64%. The values for Jurk4 are even lower, with *Novacetimonas hansenii* NBRC 14820^T^ being its closest neighbor, having an ANIb value of 88.64% and an ANIm value of 90.33%. ANI values must be lower than 95% to assert that compared strains belong to different species [41,52,53,54]. The novel species status of both strains, Hr1 and Jurk4, was also confirmed by in silico DNA-DNA hybridization calculations (Table S2). Hr1 showed ≤51.8% identity to type strains of species of the *Komagataeibacter* genus, and Jurk4 showed ≤38.6% identity to type strains of species of the *Novacetimonas* genus. These values are lower than 70%, which is the recommended cut-off point for species delineation [55].

### 3.4. Results of Phenotypic Analysis

The strain Hr1 can be differentiated from type strains of *K. melomenusus, K. nataicola, K. kakiaceti* and *K. sucrofermentans* based on its ability to grow in Asai medium with D-glucose. Additionally, Hr1 produces both 2-keto-D-gluconic acid and 5-keto-D-gluconic acid, which distinguishes it from the type strain of *K. kakiaceti*, which did not make either of these acids, and from the *K. sucrofermentans* type strain, which did not produce 5-keto-D-gluconic acid. Hr1 was unable to grow in the medium with D-ribose as a sole carbon source, which distinguishes it from type strains of *K. xylinus, K. sucrofermentans*, and *K. nataicola*, which all grew in this medium. Only *K. melomenusus* grew weakly in this medium. Moreover, it was also unable to grow in the medium supplemented with sorbitol, while the other close species, *K. xylinus, K. melomenusus, K. sucrofermentans* and *K. nataicola*, grew well or weakly. Hr1 also did not grow in the medium with 1-propanol as the sole carbon source, differentiating it from the strains *K. nataicola* and *K. xylinus*. It does not grow in the presence of 30% D-glucose and on Hoyer–Frateur medium supplemented with D-glucose or ethanol. Hr1 can use nitrogen from (NH_4_)_2_SO_4_ on a Hoyer–Frateur medium supplemented with D-mannitol (Table 3).

Jurk4 produces 2-keto-D-gluconic and 5-keto-D-gluconic acid, which differentiates it from the strains *G. entanii* AV429 and the *N. pomaceti* type strain, which only produce 2-keto-D-gluconic acid. Jurk4 can be distinguished from the *N. maltaceti* type strain based on its ability to grow in a medium with glycerol as a carbon source and from *G. entanii* AV429 and the *N. hansenii* type strain based on its ability to grow in a medium with 1-propanol as a carbon source. Jurk4 does not grow on a medium supplemented with sorbitol, while all other type strains of the genus *Novacetimonas* grew well or weakly. It also did not grow in the presence of 30% glucose, distinguishing it from type strains of *N. hansenii* and *N. maltaceti*. In addition, it does not grow on Hoyer–Frateur medium supplemented with ethanol or Asai medium supplemented with D-glucose or ethanol.

The cellular fatty acid profiles of the strains Hr1 and Jurk4 were analyzed from cells cultured on an RAE medium with 1% (*v*/*v*) acetic acid and 1% (*v*/*v*) ethanol (Table S3). The major cellular fatty acid was C_18:1_ *ω*7*c* (cis-vaccenic acid), accounting for 50.12% of the fatty acid content for Hr1 and 59.02% of the fatty acid content for Jurk4. Cis-vaccenic acid is a typical fatty acid for AAB. The second most predominant fatty acid in Hr1 was 2-hydroxy palmitic acid (C_16:0_ 2-OH), accounting for 12.04%, and palmitic acid, accounting for 11.27% of the fatty acid content in Jurk4. The amounts of C_14:0_, C_14:0_ 2-OH, and C_16:0_ 2-OH were significantly lower in the strain Jurk4 compared to Hr1.

In general, AAB are considered safe organisms. However, horizontal gene transfer may occur from AAB to pathogenic bacteria in the human gut through food consumption, for instance, potentially introducing genes for antibiotic resistance. Therefore, it is important to assess the antibiotic resistance of AAB. Both strains were resistant to gentamicin, ampicillin, ciprofloxacin, erythromycin, and trimethoprim on an RAE medium with 1% (*v*/*v*) ethanol and 1% (*v*/*v*) acetic acid. Jurk4 showed inhibition zones around chloramphenicol, while Hr1 resisted it. Using the Comprehensive Antibiotic Resistance Database [39], we searched genomes of the strains Hr1 and Jurk4 for possible antibiotic resistance molecular determinants with an online Resistance Gene Identifier. Both strains contained the *qacJ* gene. The *qacJ* gene confers an efflux mechanism and resistance to disinfectants and antiseptics [57,58] and might also be correlated with the resistance of Hr1 and Jurk4 to antibiotics.

### 3.5. Genome Analysis and Exopolysaccharide Production

During vinegar production, AAB convert ethanol to acetic acid through two sequential catalytic reactions involving membrane-bound pyrroloquinoline-quinone-dependent alcohol dehydrogenase (PQQ-ADH) and membrane-bound aldehyde dehydrogenase (ALDH) [59,60,61]. Hr1 and Jurk4 have a single homologous copy of the PQQ-ADH catalytic subunit (AdhA) in their genomes, AB1301_11805 and AB1302_15850, respectively, which is in line with previous studies on *Komagataeibacter* species [37]. Additionally, both Hr1 and Jurk4 also contain ALDH in their genomes, AB1301_06310 and AB1302_06230, respectively. In addition to PQQ-ADH, other factors have been identified that enhance bacterial resistance to acetic acid, such as succinyl-coenzyme A (CoA), an acetate CoA transferase encoded by the *aarC* gene, which is part of the modified citric acid responsible for the assimilation of acetic acid [62]. Hr1 and Jurk4 possess the *aarC* gene, AB1301_05870 and AB1302_05750, respectively.

AAB can also actively pump acetic acid from the cytosol to the periplasmic space. Homologs of H^+^ antiporters Tpo2, Tpo3, Aqr1, and Azr1 may play a role in acetic acid resistance [63]. Hr1 and Jurk4 code for homologs of Azr1, AB1301_03805, and AB1302_07485, respectively.

AAB produce various polysaccharides, including structural, intracellular, and extracellular polysaccharides. Extracellular polysaccharides belong to two types: capsular polysaccharides (CPSs) and noncapsular exopolysaccharides (EPSs). CPSs are attached to the cell surface, while EPSs are released on the cell surface without attachment [64]. Capsular polysaccharides of AAB form biofilms that help retain bacterial cells on the culture surface, which is important since the AAB are strict aerobes. The genes responsible for CPS synthesis are *rfbABCD* [65] and rfbA-rfbD homologs are also present in Hr1 (AB1301_10570, AB1301_10575, AB1301_10670, AB1301_10675) and Jurk4 (AB1302_11785, AB1302_11780, AB1302_12115, and AB1302_12120).

**Figure 4:**
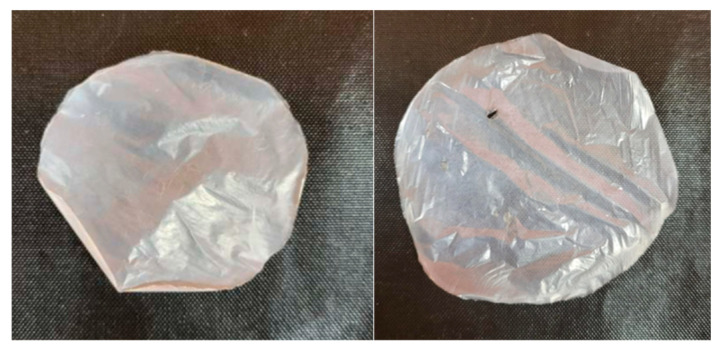
Air-dried cellulose membranes produced by *K. piraceti* Hr1 (**left**) and *N. labruscae* Jurk4 (**right**).

Extracellular polysaccharides have functions in microbial attachment, biofilm formation, and desiccation protection, with a wide range of applications in food, pharmacy, and biomedical products due to their biodegradability and unique properties. Cellulose synthesis is a characteristic of the genera *Komagataeibacter* and *Novacetimonas* [1,66,67,68,69]. Both Hr1 and Jurk4 produce cellulose (Figure 4) but differ in their production yield, with 1.56 g/L for Hr1 and 0.74 g/L for Jurk4. The surface area of the Jurk4 membrane was 66.4% larger than the surface area of the membrane synthesized by the strain Hr1. The reason for the increased surface area of the membrane will need to be further investigated. However, it may lie in differences in the operon structure for cellulose synthesis.

**Figure 5:**
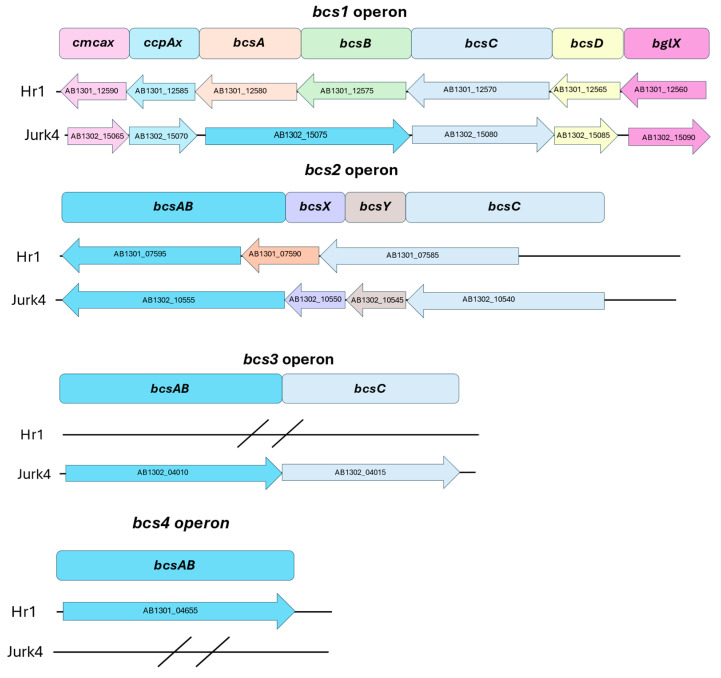
Structure of operons coding for cellulose production (*bcs1*, *bcs2*, *bcs3,* and *bcs4*) in genomes of *K. piraceti* Hr1 and *N. labruscae* Jurk4. The gene sizes are represented relative to each other. Locus tags are given in the arrows.

The genomes of Hr1 and Jurk4 were further analyzed for their characteristic operons and gene clusters. It has previously been shown that the members of the *Novacetimonas* genus possess a *bcsABCD* operon different from that of *Komagataeibacter* [1]. This *bcs1* operon encodes the main enzyme complex, while *bcs2* encodes likely an acetylated polymer. It likely also affects its attachment to the cell surface and cellulose fibers [70]. Jurk4 contains three different operons (*bcs1*, *bcs2*, and *bcs3*). In all operons, the genes *bcsA* and *bcsB* are fused into *bcsAB*, as shown in Figure 5. However, in *bcs1*, both genes are truncated for 30 amino acids (AA). The strain Hr1 also possesses three operons: *bcs1, bcs2,* and *bcs4.* In the operon *bcs2*, the genes *bcsA* and *bcsB* are fused with an internal stop. Also, *bcsX* and *bcsY* are fused but truncated; only 108 AA out of 607 remain (Figure 5). The exact function of the operons *bcs3* and *bcs4* is unknown. Differences in the cellulose membranes of the strains Jurk4 and Hr1 may thus be due to Jurk4 containing the operon *bcs3* but lacking the operon *bcs4*, while the situation is reversed in Hr1.

The genes responsible for acetan-like polysaccharides were identified in both strains (Figure S1). All species from the *Komagataeibacter* genus possess at least one acetan genetic cluster [71]; therefore, it was not surprising that genes for synthesizing acetan-like polysaccharides were found in strain Hr1. Hr1 contains a gene cluster for acetan synthesis type I, while Jurk4 has a gene cluster for acetan synthesis type II (Figure S1). The isolation of acetans from these two species posed a significant challenge, as both strains produced strong bacterial nanocellulose, into which the acetan was likely integrated. It is known that water-soluble extracellular polysaccharides can be closely associated with cellulose fibers [21].

The genomes were also analyzed for the presence of genes coding for levansucrase. These genes were not found in Hr1 and Jurk4. However, Jurk4 possessed genes for levanase. Levansucrase is an enzyme for levan synthesis, with high affinity for sucrose and low affinity for fructose and glucose [72]. Levanase, on the other hand, causes the hydrolysis of levan [73].

Since it is crucial to use multiple bioinformatic tools to capture the full spectrum of prophage diversity, the distribution of prophages in both Jurk4 and Hr1 genomes was evaluated by means of PHASTEST and Cenote-Taker 2 (Table 4 and Table 5). PHASTEST identified two prophages in each strain, among which there was only one intact prophage, present in Jurk4. Cenote-Taker 2 identified one prophage in Hr1 that spans across the two separate regions detected by PHASTEST. This suggests that the prophage identified by Cenote-Taker 2 may represent a larger, continuous prophage structure that PHASTEST classified separately. Besides this prophage, Cenote-Taker 2 identified another region as a prophage in Hr1. Both prophages identified in Hr1 by Cenote-Taker 2 are near the operon *bcs2*. In the case of Jurk4, both Cenote-Taker 2 and PHASTEST identified the same prophages.

**Figure 6:**
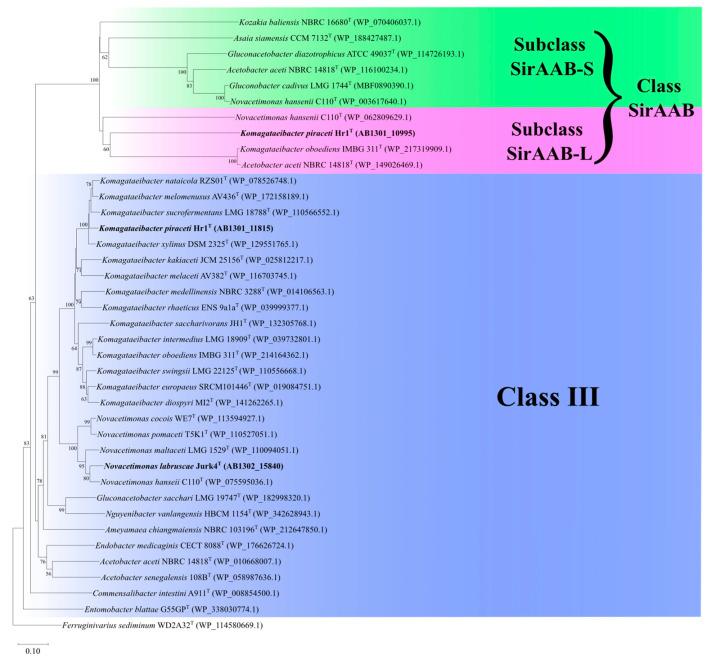
Phylogenetic reconstruction based on 39 amino acid sequences of sirtuins of the AAB. The tree was constructed using the neighbor-joining method. The newly identified sirtuins are written in bold text. The clades are divided into two sirtuin classes: Class III (blue color) and Class SirAAB (green and pink color). Class SirAAB is further divided into Subclass SirAAB-S (green color) and Subclass SirAAB-L (pink color). Bootstrap values are indicated at branching (1000 replicates). The scale bar represents 10% of estimated sequence differences.

In the annotated GenBank files of the *K. piraceti* Hr1 and *N. labruscae* Jurk4 genomes, two sirtuins were identified in the Hr1 strain and one in the Jurk4 strain. Their amino acid sequences were further analyzed using InterProScan. The analysis confirmed that the sirtuins with the locus tags AB1301_11815 (Hr1) and AB1302_15840 (Jurk4) function as NAD^+^-dependent deacylases, whereas the sirtuin with the locus tag AB1301_10995 (Hr1) could not be further annotated. According to InterProScan, both annotated proteins belong to the Sirtuin Class III family (IPR027546). Key residues characteristic of this class are present in the two newly found sirtuins, including the NAD^+^-binding site, the zinc-binding site containing four cysteine residues, and the active site responsible for proton exchange. Besides the classic NAD^+^-binding and NAD^+^-dependent deacylation activities, these sirtuins may also possess additional molecular functions, such as protein-malonyllysine demalonylase activity (GO:0036054) and protein-succinyllysine desuccinylase activity (GO:0036055). The position of the sirtuin-like proteins in *K. piraceti* Hr1 and *N. labruscae* Jurk4, among other proteins, across different genera of AAB is presented in Figure 6.

## 4. Conclusions

### 4.1. Description of Komagataeibacter piraceti sp. nov.

*Komagataeibacter piraceti* (pi.ra.ce’ti. L. n. pirum, pear; L. n. acetum, vinegar; N.L. n. *piraceti*, of pear vinegar, from where the type strain was isolated) cells are Gram-negative, rod-shaped, and have an average length of 2.35 µm and an average width of 0.64 µm. Colonies are beige, circular, and smooth, with diameters of about 0.5 mm on RAE medium supplemented with 1% (*v*/*v*) ethanol and 1% (*v*/*v*) acetic acid after three days of incubation at high relative humidity. Cells grow in aerobic conditions, but also in an atmosphere with lower concentrations of oxygen. Cells are catalase-positive and oxidase-negative. Ethanol is oxidized to acetic acid and further to CO_2_ and H_2_O. Both 2-keto-D-gluconic and 5-keto-D-gluconic acid are formed. Growth is present in 1–3% acetic acid and 1% ethanol and 1–2% acetic acid and 3% ethanol, but acetic acid is not required for growth. Growth is observed on mannitol agar and glucose–yeast agar. Weak growth is observed in D-gluconate, glycerol, D-mannitol. The strain can utilize ammoniacal nitrogen in Hoyer–Frateur medium with D-mannitol but not with ethanol and D-glucose, and in Asai medium with D-glucose and D-mannitol but not with ethanol. C_18:1_ *ω*7*c* (cis-vaccenic acid) is the most abundant fatty acid, followed by 2-hydroxy palmitic acid (C_16:0_ 2-OH). The strain produces cellulose, which makes it industrially valuable.

The type strain Hr1^T^ (=ZIM B1167^T^ = LMG 33628^T^) was isolated from pear vinegar in Pohorje, Slovenia.

The GenBank/ENA/DDBJ accession numbers for the complete genome sequence are CP160616-CP160619. The accession numbers for the 16S-23S rRNA gene sequence ITS and the 16S rRNA gene sequence are PQ641081 and PV083163, respectively.

### 4.2. Description of Novacetimonas labruscae sp. nov.

*Novacetimonas labruscae* (la.brus’cae. L. n. *labrusca*, wild vine or grape; N.L. n. *labruscae*, from *Vitis labrusca*, indicating its origin from vinegar made with apples and grapes of the *Vitis labrusca* variety) cells are Gram-negative and have an average length of 3.02 µm and an average width of 0.89 µm. The shapes of colonies are irregular, with sizes around 0.5–1 mm in diameter after three days of cultivation on RAE medium supplemented with 1% (*v*/*v*) acetic acid and 1% (*v*/*v*) ethanol in high air humidity. Microscopical examinations showed small rods. Cells grow in aerobic conditions, but also in atmospheres with lower concentrations of oxygen. Cells are catalase positive and oxidase negative. Ethanol is oxidized to acetic acid and further to CO_2_ and H_2_O. Bacterial cellulose is produced from D-glucose. Growth is observed in 1–3% acetic acid and 1 or 3% ethanol, but acetic acid is not required for growth. Growth is observed on glucose–yeast agar and mannitol agar. Growth is observed on D-mannitol and glycerol and weak growth is observed with 1-propanol and D-ribose as a sole carbon source. The strain can weakly utilize ammoniacal nitrogen in Hoyer–Frateur medium with D-glucose and D-mannitol, but not ethanol, and in Asai medium with D-mannitol, but not D-glucose and ethanol. C_18:1_ *ω*7*c* (cis-vaccenic acid) is the most abundant fatty acid, while the second most abundant fatty acid is palmitic acid. The strain produces cellulose, which makes it industrially valuable.

The type strain Jurk4^T^ (=ZIM B1166^T^ = LMG 33630^T^) was isolated from vinegar made with apples and grapes in Pohorje, Slovenia.

The GenBank/ENA/DDBJ accession numbers for the complete genome sequence are CP160612-CP160615. The accession numbers for the 16S-23S rRNA gene sequence ITS and the 16S rRNA gene sequence are PQ641080 and PV083162, respectively.

## Figures and Tables

**Table 1:** Data on basic chemical characteristics of pear and apple-grape vinegars.

	Pear Vinegar	Apple-Grape Vinegar
Titratable acids (% *w/v*)	0.54	3.86
pH	4.14	3.33
Alcohol content (% *v*/*v*)	1.00	0.30
Reducing sugars (% *w/v*)	3.80	0.00

**Table 2:** List of AAB strains isolated from pear and apple-grape vinegars.

Pear Vinegar	Apple-Grape Vinegar
*Acetobacter aceti* Hrc1	*Komagataeibacter xylinus* Jurk1
*Acetobacter persici* Hrc2	*Novacetimonas labruscae* Jurk4
*Komagataeibacter piraceti* Hr1	*Acetobacter aceti* Jurka2
*Acetobacter* sp. nov. Hr2	
*Acetobacter ghanensis* Hr-1-1	
*Acetobacter musti* Hr-1-4	
*Gluconacetobacter* sp. nov. Hr-1-5	
*Acetobacter pasteurianus* Hr-1-6	
*Komagataeibacter xylinus* Hr-3-4	

**Table 3:** Phenotypic characteristics of *Komagataeibacter* strain Hr1 and *Novacetimonas* strain Jurk4 in comparison to other *Komagataeibacter* and *Novacetimonas* species. Species: 1, Hr1; 2, *K. melomenusus* AV436^T^; 3, *K. xylinus* LMG 1515^T^; 4, *K. nataicola* LMG 1536^T^; 5, *K. kakiaceti* LMG 26206^T^; 6, *K. sucrofermentans* LMG 18788^T^; 7. Jurk4, 8. *N. cocois* JCM 31140^T^; 9, *G. entanii* AV429; 10, *N. hansenii* LMG 1527^T^; 11, *N. pomaceti* T5K1^T^; 12, *N. maltaceti* LMG 1529^T^. Data marked with letters were obtained from the following publications: ^a^, Castro et al. [56]; ^b^, Marič et al. [38]; ^c^, Škraban et al. [37].

Phenotypic Features	1	2	3	4	5	6	7	8	9	10	11	12
Formation from D-glucose:												
2-Keto-D-gluconic acid	+	+	+ ^a^	+	−	+	+	+	+	+	+	+
5-Keto-D-gluconic acid	+	+	+ ^a^	+	−	−	+	+	−	+	−	+
Growth on carbon sources:												
D-Ribose	−	w	+ ^b^	+	+	+	w	+	+	+	+	w
Sorbitol	w	w	+ ^b^	+	+	−	−	w	+	+	w	w
Glycerol	−	w	+ ^b^	+	−	−	+	+	+	+	w	−
D-Gluconate	w	−	+ ^c^	+	−	−	−	w	−	w	w	−
1-Propanol	−	−	+ ^b^	+	−	−	+	w	−	−	w	+
Growth in the presence of 30% D-glucose	w	−	− ^b^	+	w	−	−	−	w	+	−	+
Utilization of ammoniacal nitrogen in:												
Hoyer–Frateur medium with:												
D-Glucose	−	+	nd	w	+	w	w	+	+	+	−	w
D-Mannitol	+	+	nd	+	−	+	w	+	+	+	−	+
Ethanol	−	−	+ ^b^	+	−	−	−	w	+	+	−	+
Asai medium with:												
D-Glucose	+	−	nd	−	−	−	−	+	+	+	−	w
Ethanol	w	−	nd	−	−	−	−	w	+	+	−	−

+, positive; −, negative; w, weak reaction; nd, no data available.

**Table 4:** Prophage identification in the genomes of *K. piraceti* Hr1 and *N. labruscae* Jurk4 using the web-based tool PHASTEST.

Bacterial Host Strain	Region Length (kb)	Completeness	Number of ORFs Present in Region	Region Position
Hr1	23.3	questionable	37	1618846–1642228
	13.7	questionable	16	1643155–1656881
Jurk4	49.0	Intact	150	398088–447149
	20.7	questionable	70	2189361–2210127

**Table 5:** Prophage identification in the genomes of *K. piraceti* Hr1 and *N. labruscae* Jurk4 using the Cenote-Taker 2 bioinformatic tool.

Bacterial Host Strain	Region Length (kb)	Region Position	Viral Hallmark Genes
Hr1	42.0	1662600–1704600	3
	39.0	1616350–1655350	11
Jurk4	51.1	396800–447850	14
	32.2	2186200–2218400	10

## Data Availability

The original contributions presented in the study are included in the article/Supplementary Material, further inquiries can be directed to the corresponding author.

## Supplementary Materials

The following supporting information can be downloaded at: https://www.mdpi.com/article/10.3390/microorganisms13020456/s1, Table S1: ANIb and ANIm analysis of complete genomes of Hr1 and Jurk4 compared to other *Komagataeibacter* and *Novacetimonas* type species. Table S2: In silico DNA–DNA hybridization (dDDH) analysis of complete genomes of Hr1 and Jurk4 compared to other *Komagataeibacter* and *Novacetimonas* type species. Table S3: Cellular fatty acid profiles of the strains Hr1 and Jurk4. Figure S1: Schematic structure of two types of acetan clusters in Hr1 and Jurk4 genomes. The gene sizes are shown relative to each other.
