# Supplementary material for: Isolation and Characterization of Komagataeibacter piraceti sp. nov. and Novacetimonas labruscae sp. nov.: Two Novel Microaerobic Cellulose-Producing Acetic Acid Bacteria from Vinegars
Source: Microorganisms. 2025 Feb 19;13(2):456. doi: 10.3390/microorganisms13020456 (PMC11858473; doi:10.3390/microorganisms13020456)
Supplement: Supplementary file 1 [file microorganisms-13-00456-s001.zip › microorganisms-3455466-supplementary.pdf]

Karničnik et al.: Isolation and Characterization of *Komagataeibacter piraceti* sp. nov. and *Novacetimonas labruscae* sp. nov.: Two Novel Microaerobic Cellulose-Producing Acetic Acid Bacteria from Vinegars

Supplementary Materials

Table S1: ANIb and ANIm analysis of complete genomes of Hr1 and Jurk4 compared to other *Komagataeibacter* and *Novacetimonas* type species.

	**ANIb Jurk4**	**ANIm Jurk4**
Novacetimonas hansenii NBRC 14820	88.64	90.33
Gluconacetobacter entanii LTH 4560	88.33	90.01
Novacetimonas maltaceti LMG 1529	87.83	89.80
Novacetimonas pomaceti T5K1	85.02	88.07
Novacetimonas cocois WE7	84.03	87.39
	**ANIb Hr1**	**ANIm Hr1**
Komagataeibacter melomenusus AV436	92.62	93.64
Komagataeibacter xylinus LMG 1515	91.45	92.67
Komagataeibacter sucrofermentans LMG 18788	91.15	92.35
Komagataeibacter nataicola LMG 1536	90.51	91.97
Komagataeibacter europaeus LMG 18890	84.17	87.56
Komagataeibacter swingsii LMG 22125	84.09	87.34
Komagataeibacter oboediens LMG 18894	83.89	87.28
Komagataeibacter intermedius LMG 18909	83.73	87.21
Komagataeibacter melaceti AV382	83.15	86.98
Komagataeibacter kakiaceti JCM 25156	82.98	86.93
Komagataeibacter rhaeticus LMG 22126	82.88	86.63
Komagataeibacter medellinensis NBRC 3288	82.67	87.07
Komagataeibacter diospyri MSKU9	82.16	86.01
Komagataeibacter saccharivorans LMG 1582	81.13	85.56

Table S2: *In-silico* DNA-DNA hybridization (dDDH) analysis of complete genomes of Hr1 and Jurk4 compared to other *Komagataeibacter* and *Novacetimonas* type species.

	**Jurk4 (%)**
Novacetimonas hansenii JCM 7643	38.6
Novacetimonas hansenii NBRC 14820	38.5
Komagataeibacter kombuchae LMG 23726	38.5
Gluconacetobacter entanii LTH 4560	37.3
Novacetimonas maltaceti LMG 1529	36.6
Novacetimonas pomaceti T5K1	30.7
Novacetimonas cocois WE7	29.2
Komagataeibacter swingsii LMG 22125	26.6
Komagataeibacter europaeus LMG 18890	26.4
Komagataeibacter intermedius LMG 18909	26.1
Komagataeibacter xylinus NBRC 15237	25.9
	**Hr1 (%)**
Komagataeibacter melomenusus AV436	51.8
Komagataeibacter xylinus NBRC 15237	47.1
Komagataeibacter sucrofermentans LMG 18788	46.4
Komagataeibacter nataicola LMG 1536	44.7
Komagataeibacter europaeus LMG 18890	30.3
Komagataeibacter swingsii LMG 22125	29.3
Komagataeibacter oboediens LMG 18849	29.2
Komagataeibacter intermedius LMG 18909	29.0
Komagataeibacter melaceti AV382	28.0
Komagataeibacter medellinensis NBRC 3288	27.7
Komagataeibacter rhaeticus LMG 22126	27.5
Komagataeibacter diospyri MSKU 9	26.5

Table S3: Cellular fatty acid profiles of the strains Hr1 and Jurk4.

Fatty acid	Hr1 (%)	Jurk4 (%)
$C_{12:0}$	1.12	0.53
$C_{14:0}$	8.45	4.21
$C_{16:0}$	9.28	11.27
$C_{17:0}$	Nd	1.56
$C_{18:0}$	1.99	2.26
$C_{20:0}$	0.45	1.18
$C_{18:1}$ $\omega7c$	50.12	59.02
$C_{14:0}$ 2-OH	6.95	4.97
$C_{16:0}$ 2-OH	12.04	8.31
$C_{16:0}$ 3-OH	3.87	2.11
$C_{18:0}$ 3-OH	1.05	1.15
$C_{16:1}$ $\omega7c$/$C_{16:1}$ $\omega6c$	1.16	0.60
$C_{14:0}$ 3OH/ $C_{16:1}$ iso I	1.45	1.02
$C_{19:0}$ cyclo $\omega8c$	1.33	0.27

Nd, not detectable

Figure S1. Schematic structure of two types of acetan clusters in strains Hr1 and Jurk4 genomes. The gene sizes are shown relative to each other.

Acetan cluster type I

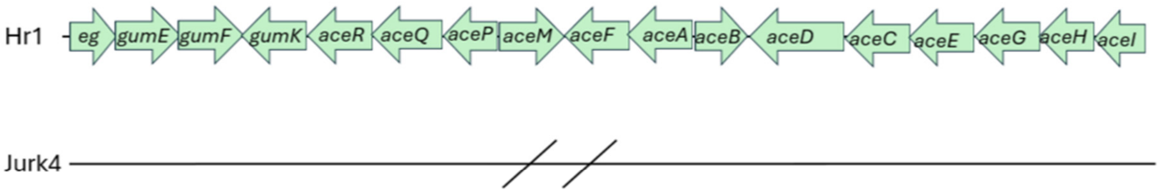

ID	Encodes	Locus tag Hr1
eg	glycoside hydrolase (family 5 protein)	AB1301_10665
gumE	polymerization or export protein	AB1301_10660
fumF	acetyltransferase	AB1301_10655
gumK	glucuronosyl transferase	AB1301_10650
aceR	rhamnosyl transferase	AB1301_10645
aceQ	glucosyl transferase	AB1301_10640
aceP	glucosyl transferase	AB1301_10635
aceM	UDP-glucose/ GDP-mannose dehydrogenase	AB1301_10630
aceF	mannose-1-phosphate guanylyltransferase/ mannose-6-phosphate isomerase	AB1301_10625

ID	Encodes	Locus tag Hr1
aceA	exopolysaccharide biosynthesis polyprenyl glycosylphotransferase	AB1301_10620
aceB	glucosyl transferase	AB1301_10615
aceD	polymerization and export protein	AB1301_10610
aceC	mannose transferase	AB1301_10605
aceE	flippase	AB1301_10600
aceG	outer membrane beta-barrel protein	AB1301_10595
aceH	polysaccharide export protein	AB1301_10690
aceI	acyltransferase	AB1301_10685

Acetan cluster type II

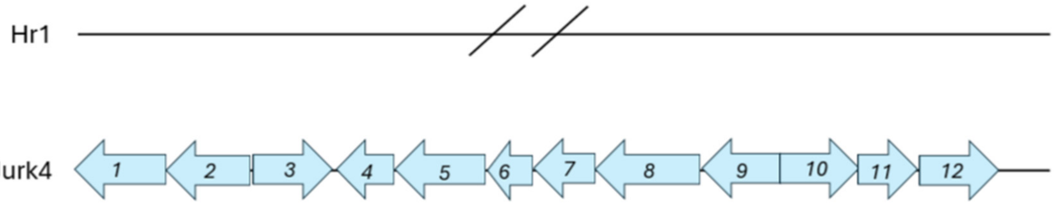

ID	Encodes	Locus tag Jurk4
1	flippase	AB1302_01150
2	galE UDP-glucose 4-epimerase	AB1302_01155
3	acyltransferase	AB1302_01160
4	glycosyltransferase (family 2)	AB1302_01165
5	glycosyltransferase	AB1302_01170
6	hypothetical protein	AB1302_01175

ID	Encodes	Locus tag Jurk4
7	glycosyltransferase (family 4)	AB1302_01180
8	polysaccharide biosynthesis tyrosine	AB1302_01185
9	biosynthesis polyprenyl glycosylphosphotransferase	AB1302_01190
10	outer membrane beta-barrel protein	AB1302_01195
11	polysaccharide export protein	AB1302_01200
12	glycosyltransferase	AB1302_01210
